# Continued Circulation of Tick-Borne Encephalitis Virus Variants and Detection of Novel Transmission Foci, the Netherlands

**DOI:** 10.3201/eid2812.220552

**Published:** 2022-12

**Authors:** Helen J. Esser, Stephanie M. Lim, Ankje de Vries, Hein Sprong, Dinant J. Dekker, Emily L. Pascoe, Julian W. Bakker, Vanessa Suin, Eelco Franz, Byron E.E. Martina, Constantianus J.M. Koenraadt

**Affiliations:** Wageningen University and Research, Wageningen, the Netherlands (H.J. Esser, D.J. Dekker, E.L. Pascoe, J.W. Bakker, C.J.M. Koenraadt);; Artemis One Health Research Institute, Delft, the Netherlands (S.M. Lim, B.E.E. Martina);; National Institute for Public Health and the Environment, Bilthoven, the Netherlands (A. de Vries, H. Sprong, E. Franz);; Sciensano, Brussels, Belgium (V. Suin)

**Keywords:** tick-borne encephalitis virus, tick-borne encephalitis, *Ixodes ricinus*, rodent, human cases, antibodies, monitoring, surveillance, sentinel, tickborne disease, distribution, emergence, arbovirus, *Apodemus*, *Myodes*, *Microtus*, vector-borne infections, viruses, the Netherlands

## Abstract

Tick-borne encephalitis virus (TBEV) is an emerging pathogen that was first detected in ticks and humans in the Netherlands in 2015 (ticks) and 2016 (humans). To learn more about its distribution and prevalence in the Netherlands, we conducted large-scale surveillance in ticks and rodents during August 2018–September 2020. We tested 320 wild rodents and >46,000 ticks from 48 locations considered to be at high risk for TBEV circulation. We found TBEV RNA in 3 rodents (0.9%) and 7 tick pools (minimum infection rate 0.02%) from 5 geographically distinct foci. Phylogenetic analyses indicated that 3 different variants of the TBEV-Eu subtype circulate in the Netherlands, suggesting multiple independent introductions. Combined with recent human cases outside known TBEV hotspots, our data demonstrate that the distribution of TBEV in the Netherlands is more widespread than previously thought.

Tick-borne encephalitis (TBE) is one of the most frequently occurring arboviral diseases in Europe and Asia; 10,000–15,000 human cases occur each year (*1*). TBE-endemic regions of Europe experienced a 400% increase in the number of cases during 1973–2003, but the notification rate has remained relatively stable over the past 2 decades (with the exception of some peak years, such as 2006 and 2018) (*2*,*3*). On the local scale, however, marked fluctuations in disease incidence have occurred over time (*3*). Ecologic, climatic, socioeconomic, and cultural aspects might all play a role in explaining these dynamics, but their relative importance might vary across TBE-endemic regions (*4*–*6*). Transmission of TBE virus (TBEV) is dependent on complex ecologic interactions between TBEV, tick vectors (in Europe, principally *Ixodes ricinus*) and vertebrate reservoir hosts (small rodents of the genera *Apodemus*, *Myodes*, and *Microtus*) and appears to occur only under specific environmental conditions (*7*). As a result, the occurrence of TBEV is characterized by a scattered and strongly focal pattern, despite the widespread occurrence of both vector and reservoir hosts (*7*).

Of note, new endemic TBEV foci continue to emerge, both in countries where the virus has been present for a long time (e.g., Germany, Czech Republic, and Baltic states) and in countries where it was considered absent (e.g., the Netherlands and United Kingdom) (*3*,*8*,*9*). The recent detection of TBEV in previously unaffected countries indicates that the current distribution of the virus lies beyond what was predicted by past climate suitability models (*10*). The mechanisms underlying this unexpected emergence remain unclear and underline the need for systematic data collection on virus prevalence in emerging areas.

The Netherlands was long considered a nonendemic country for TBEV because human TBE cases were all associated with travel (*11*) and past surveillance studies did not find evidence of virus circulation in local wildlife or ticks (*12*). This situation changed in 2015, when TBEV was first detected in ticks collected in response to retrospective serologic screening of serum samples from roe deer (*Capreolus capreolus*), which indicated the virus might have been circulating in the Netherlands as far back as 2010 (*13*). A follow-up study also using roe deer as sentinel hosts suggested that the spatial distribution of the virus had increased by 2017 (*14*). Yet TBEV RNA–positive ticks and autochthonous human TBE cases had until then been reported from just 2 nature areas: National Park de Utrechtse Heuvelrug in the municipality of Zeist and National Park de Sallandse Heuvelrug in the municipalities of Hellendoorn and Rijssen-Holten (*9*). Thus, local circulation of TBEV in the potential foci identified by serologic screening of roe deer required confirmation. This need prompted us to undertake large-scale surveillance of ticks and wild rodents to investigate TBEV presence and prevalence in potential new foci in the Netherlands.

## Materials and Methods

### Sample Collection

**Figure 1:**
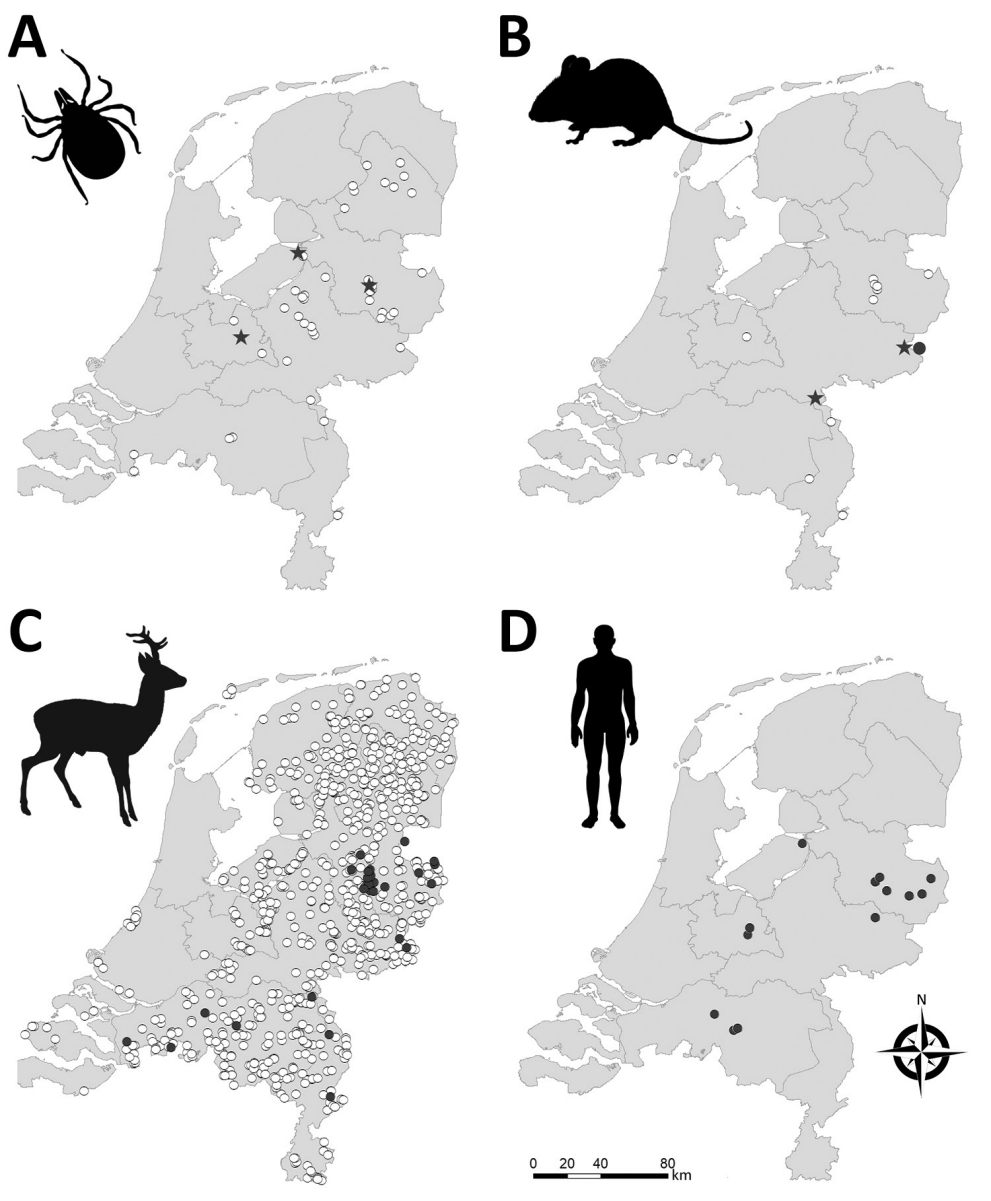
Geographic distribution of tick-borne encephalitis virus (TBEV) in the Netherlands based on sampling of ticks (A), rodents (B), roe deer (C), and reported human (D) tick-borne encephalitis cases. Stars indicate TBEV RNA–positive tick pools or rodent samples. Closed circles indicate serum samples that tested positive in TBEV serum neutralization tests. White circles indicate negative test results. Data for roe deer were reproduced from Rijks et al. (*14*) with permission. Maps were constructed with Arc-GIS software (ESRI, https://www.esri.com).

We collected >46,000 questing ticks (3,321 adult females, 3,764 adult males, and 39,025 nymphs) by drag sampling in 46 locations in September 2018 and during March–June 2019 and April–September 2020 (Figure 1). In addition, we collected 320 rodents and 1,370 ticks feeding on those rodents (1,342 larvae and 28 nymphs) from 13 locations during August–October 2018 and March–June 2019 (Figure 1). All but 2 of the rodent sampling locations coincided with the 46 drag sampling locations. Thus, in total, we sampled 48 locations for questing ticks, rodents, or both. Sampling locations were all in forested nature areas throughout the Netherlands located as close as possible to places where seropositive roe deer were detected in Rijks et al. (*14*) or where the environmental suitability for TBEV circulation was highest according to Esser et al. (*15*). One location, however, involved the woodland garden of an autochthonous TBE patient, where TBEV RNA–positive ticks had been collected in 2017 and 2018 (*9*). That garden borders National Park de Sallandse Heuvelrug and lies within 2 km of the location where TBEV RNA–positive ticks and seropositive roe deer were found in 2016 (*13*). We refer to this garden as Nijverdal garden. We obtained research clearance from all terrain owners to collect ticks, rodents, or both.

At each location, we trapped rodents by using Heslinga live traps that were placed in a 7 × 7 grid with 10 meters’ interspacing between traps. We filled traps with hay and baited them with a mixture of grains, carrots, and mealworms. We transported captured rodents to the laboratory facility, where we took blood samples from the submandibular vein under isoflurane anesthesia and subsequently euthanized the animals by cervical dislocation. We identified collected rodents to the species level by morphologic and molecular methods (*16*); the specimens consisted of *Apodemus flavicollis* mice (n = 29), *A. sylvaticus* mice (n = 199), *Microtus arvalis* voles (n = 2), and *Myodes glareolus* voles (n = 90). We collected brain and visceral organ tissues from each rodent and all feeding ticks, if present, and stored samples at −80°C until further analysis. All handling procedures were approved by the Animal Experiments Committee of Wageningen University (approval nos. 2017.W-0049.003 and 2017.W-0049.005) and by the Netherlands Ministry of Economic Affairs (approval no. FF/75A/2015/014).

### TBEV RNA Detection and Tick Species Identification

We transported ticks collected from vegetation alive to the laboratory and pooled (4 females/pool, 8 males/pool, or 25 nymphs/pool) by sampling location. However, we tested ticks collected from the Nijverdal garden (37 females, 57 males, and 1,100 nymphs) individually, because we expected this location to have the highest probability of harboring TBEV-infected ticks. We homogenized ticks and extracted nucleic acid as previously described (*17*). To obtain sequences of real-time quantitative reverse transcription PCR (qRT-PCR)–positive tick samples, we performed conventional PCR targeting the polyprotein region of the virus by using primers and protocols as previously described (*18*), then performed sequencing.

In the laboratory, we removed ticks alive from rodents and pooled them per rodent (<3 nymphs/pool or <50 larvae/pool). However, we tested ticks collected from TBEV RNA–positive rodents (33 larvae in total) individually. We identified tick species by using a TaqMan qRT-PCR assay, which we also used to test the ticks for TBEV RNA (Appendix 1).

We placed small sections of spleens separately in Lysis Matrix D tubes (MPBio, https://www.mpbio.com) with added MagNa Pure 96 lysis buffer (Roche, https://www.roche.com). We performed nucleic acid extraction as described for the questing ticks. We froze half-brains −80°C in 1 ml of Dulbecco’s Modified Eagle Medium (ThermoFisher Scientific, https://www.thermofisher.com) before processing. We homogenized samples and extracted nucleic acid as described for the ticks collected from rodents and tested samples for TBEV by qRT-PCR.

### Phylogenetic Analysis

**Figure 2:**
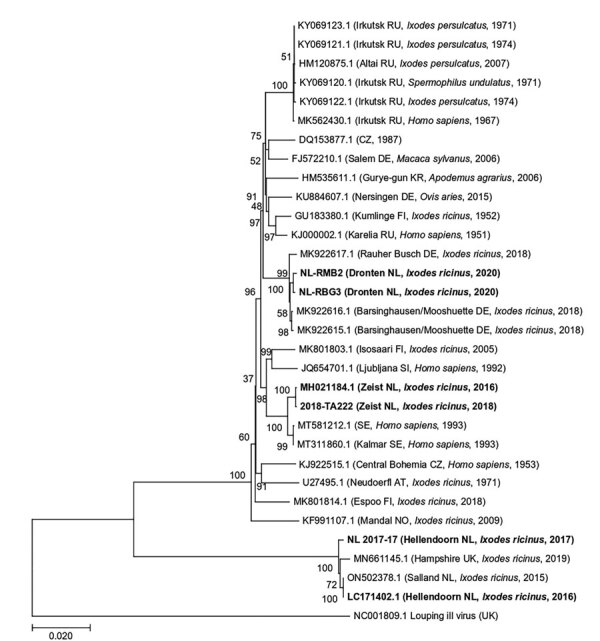
Maximum-likelihood phylogenetic tree of polyprotein sequences obtained from tick-borne encephalitis virus RNA–positive *Ixodes ricinus* ticks collected from 3 locations in the Netherlands during 2016–2020 (in bold). Additional published sequences obtained from GenBank are included for reference. Louping ill virus is used as the outgroup. Sample ID or GenBank accession numbers are indicated for each sequence, with location in brackets (if known) and country code, original isolation source, and collection year of each sample. Numbers next to each branch indicate the percentage of trees resulting from bootstrapping on the basis of 1,000 pseudoreplicate datasets for which the associated taxa clustered together. Scale bar represents the percentage of genetic variation along tree branches.

We used MEGA version 10.0.5 (https://www.megasoftware.net) to perform sequence alignments and distance matrix calculations and to construct a phylogenetic tree of polyprotein gene sequences from TBEV RNA–positive tick pools (*19*). We trimmed end-reading errors from each sequence and used BLAST (https://blast.ncbi.nlm.nih) to find and download the 10 most closely matching sequences published in GenBank (note that there was sequence repetition in BLAST results between some samples). We included sequences of the Neudoerfl strain (Genbank accession no. U27495) and Mandal strain (accession no. KF991107) for additional comparison and included Louping ill virus (accession no. NC001809) as an outgroup. We trimmed sequences to the same length (6,735 nt) and aligned by using the MUSCLE algorithm (*20*). We used the maximum-likelihood method and general time reversible model with a gamma distribution and invariant sites to construct the phylogenetic tree (*21*), as determined by jModeltest version 2.1.10 (*22*). We performed 1,000 bootstrap iterations and visualized the tree with the highest log likelihood (21093.04) (Figure 2).

### Serologic Detection in Rodents

We tested 316 rodent serum samples for antibodies against TBEV by using a commercial ELISA kit (EIA TBEV Ig; TestLine Clinical Diagnostics, https://www.testlinecd.com) optimized and verified in-house for rodents (Appendix 1). We then conducted a rapid fluorescent focus inhibition seroneutralization test on the ELISA-positive or borderline samples using TBEV Neudoerfl NCPV#848 as reference strain, as previously described (*23*). The dilution of tested serum samples that neutralizes 50% of the virus (DIL_50_) defines the seroneutralization titer. Serum samples were considered positive at DIL_50_>1/15 and negative at DIL_50_<1/10. We considered the DIL_50_ between both values doubtful.

## Results

Of the 3,086 tick pools tested (representing 44,916 questing individual ticks), 7 from 3 locations were positive for TBEV RNA (minimum infection rate 0.02%) (Table 1; Figure 1). The 1,194 individually tested ticks collected from the Nijverdal garden (municipality of Hellendoorn) were all negative for TBEV RNA. Whole-genome sequences were obtained for 3 TBEV RNA–positive tick pools: 2018-TA222 from Zeist (GenBank accession no. MZ969636) and NL-RMB2 and NL-RBG3 from Dronten (accession nos. MZ969638 and MZ969639). The 2 sequences from Dronten were 99.82% similar to one another, and the sequence from Zeist was 99.96% similar to a sequence obtained from an *I. ricinus* tick in the same municipality 2 years before (*24*). Likewise, previously obtained sequences from *I. ricinus* ticks collected in Hellendoorn in 2015 and 2017 were 99.67% similar to each other (Appendix 2). Sequence similarity between municipalities ranged from 89.45 to 97.76% and was significantly lower than similarity among ticks from the same municipality (*t* = 6.51, d.f. = 11.12; p<0.01).

**Table 1:** Pools of tick-borne encephalitis virus RNA–positive *Ixodes ricinus* ticks collected from vegetation in 3 locations in the Netherlands, 2018–2020*

Sample ID	Tick pool	Ct value	Nature area	Municipality	Year	GenBank
2018-TA222	25 nymphs	15.31	National Park Utrechtse Heuvelrug	Zeist	2018	NA
2018-TA226	25 nymphs	17.65	National Park Utrechtse Heuvelrug	Zeist	2018	MZ969636
A43	25 nymphs	19.84	National Park Utrechtse Heuvelrug	Zeist	2019	NA
4040	25 nymphs	19.42	National Park Sallandse Heuvelrug	Hellendoorn	2019	NA
NL-RGB1	4 females	29.1	Roggebotzand	Dronten	2020	NA
NL-RMB2	8 males	21.12	Roggebotzand	Dronten	2020	MZ969638
NL-RBG3	25 nymphs	17.64	Roggebotzand	Dronten	2020	MZ969639

*Ct, cycle threshold; NA, not applicable.

Phylogenetic analyses revealed that all sequences clustered within the TBEV-Eu subtype (Figure 2). When we compared sequences with other strains circulating in Europe, we found that sequences from Dronten were most closely related to the Barsinghausen/Mooshuette (Germany) strain (accession no. MK922616) isolated from ticks in 2019, sharing 99.76% (NL-RBG3) and 99.70% (NL-RMB2) sequence similarity. Sequences from Zeist (Utrechtse Heuvelrug) were most closely related to a Sweden strain isolated from a human sample (accession no. MT311860), sharing 99.52% (2018-TA222) and 99.54% (MH021184) sequence similarity. The 2 sequences from Hellendoorn (Sallandse Heuvelrug) were strongly homologous to the TBEV-NL strain previously isolated from ticks from this area (99.67% for NL2017-17 and 100% for LC171402), as well as to TBEV-UK Hampshire from ticks (MN661145), sharing 99.46% (NL2017-17) and 99.55% (LC171402) sequence similarity.

We tested for the presence of TBEV RNA in brain and spleen tissue of 320 rodents and found evidence of TBEV infection in 3 rodents (0.9%) from 2 municipalities (Table 2; Figure 1). Sequencing a fragment of the envelope protein confirmed that these variants belonged to the TBEV-Eu subtype, but the sequences were too short to perform detailed phylogenetic cluster analyses. In addition, 5 rodents tested seropositive (3 × OD_NC_ [optical density of the negative control]) and 6 were borderline (2 × OD_NC_). However, only 1 of these samples (from a *Mi. arvalis* vole) was positive in the serum neutralization test (SNT), whereas 3 had insufficient serum volume left to be confirmed by SNT (Table 2). The rodent with TBEV-neutralizing antibodies was 1 of the rodents that also tested positive for TBEV RNA.

**Table 2:** Rodents that tested positive for the presence of tick-borne encephalitis virus antibodies by SNT or viral RNA in tissue samples by PCR, the Netherlands, 2018–2020*

Sample ID	Species	Sex	Serologic result†	SNT	PCR result, Ct value	Nature area	Municipality	Year
18–2752	*Apodemus sylvaticus*	M	Weak positive	Negative	Negative	National Park de Groote Peel	Peel en Maas	2018
18–2779	*A. sylvaticus*	F	Weak positive	Not tested‡	Negative	National Park Sallandse Heuvelrug	Rijssen-Holten	2018
18–2829	*A. sylvaticus*	M	Weak positive	Negative	Negative	National Park Sallandse Heuvelrug	Hellendoorn	2018
18–2830	*A. sylvaticus*	F	Positive	Negative	Negative	National Park Sallandse Heuvelrug	Hellendoorn	2018
19–2893	*A. sylvaticus*	F	Positive	Not tested‡	Negative	National Park Utrechtse Heuvelrug	Zeist	2019
19–2895	*A. sylvaticus*	F	Positive	Negative	Negative	National Park Utrechtse Heuvelrug	Zeist	2019
19–2896	*A. sylvaticus*	M	Positive	Negative	Negative	National Park Utrechtse Heuvelrug	Zeist	2019
19–2901	*A. sylvaticus*	M	Weak positive	Negative	Negative	Springendal	Tubbergen	2019
19–2916	*A. sylvaticus*	M	Positive	Not tested‡	Negative	Nijverdal Garden	Hellendoorn	2019
19–2997	*My. glareolus*	M	Weak positive	Negative	Negative	Natuurpark de Leemputten	Oost Gelre	2019
19–3001	*Microtus arvalis*	F	Negative	Not tested	Spleen 30.74, brain 30.09	Natuurpark de Leemputten	Oost Gelre	2019
19–3002	*Mi. arvalis*	F	Weak positive	Positive	Spleen 30.57; brain 28.33	Natuurpark de Leemputten	Oost Gelre	2019
19–3053	*Myodes glareolus*	F	Negative	Not tested	Spleen 35.68; brain negative	Rijk van Nijmegen	Berg en Dal	2019

*Ct, cycle threshold; SNT, serum neutralization test. †Positive = 3 × OD_NC_ (optical density of the negative control), borderline = 2 × OD_NC_. ‡Not tested because of too little volume.

Tick infestation prevalence among rodents was high for each species: *A. flavicollis*, 96.3% (26/27); *A. sylvaticus*, 93.4% (142/152); *Mi. arvalis*, 100% (2/2); and *My. glareolus*, 66.7% (26/39). However, tick burdens varied considerably among species: *A. flavicollis*, range 0–14, median 4; *A. sylvaticus*, range 0–50, median 2; and *My. glareolus*, range 0–11, median 1. The 2 specimens of *Mi. arvalis* voles had 13 and 20 ticks. We found cofeeding between nymphs (n = 27) and larvae (n = 228) on 21 of 320 rodents (6.6%) and in 8 of 13 rodent trapping locations. In total, we tested 1,370 ticks that were removed from rodents for the presence of TBEV RNA and to determine the presence of tick species. Of the 214 tick pools tested, 192 pools contained *I. ricinus* ticks only (89.7%), 8 pools contained *I. trianguliceps* ticks only (3.7%), and 13 pools contained both species (6.1%). One tick pool was negative on both species tests, suggesting that these ticks belonged to other, unidentified tick species. Half of the tick pools that contained *I. trianguliceps* ticks were taken from *A. sylvaticus* mice (11/21) and the other half from *M. glareolus* voles (10/21). TBEV RNA was not detected in any of the tick pools collected from rodents or in the 33 individually tested larvae collected from TBEV RNA–positive rodents.

## Discussion

We conducted an intensive national screening of ticks and rodents to obtain an ecoepidemiologic picture of TBEV circulation in the Netherlands. Our results build on earlier studies (*9*,*13*,*14*,*24*) and indicate that 3 different TBEV-Eu variants cocirculate in the country. We also present evidence of epizootic transmission in the nature areas of Roggebotzand (municipality of Dronten), Rijk van Nijmegen (Berg en Dal), and Natuurpark de Leemputten (Oost-Gelre), which are all located outside the known TBEV hotspots Utrechtse Heuvelrug (Zeist) and Sallandse Heuvelrug (Hellendoorn and Rijssen-Holten). Together with recent human cases in several municipalities where clinical TBE had thus far not been reported (Figure 1), these findings suggest that the distribution of TBEV in the Netherlands is more widespread than previously found.

We found a significantly lower phylogenetic similarity between TBEV sequences from questing ticks at different municipalities compared with sequences from the same municipality. In specific, whole-genome sequences from Dronten, Zeist, and Hellendoorn were more closely related to strains from Germany, Sweden, and England, respectively, than to each other. These findings are in line with other studies from elsewhere in Europe, which also found high genetic diversity among local TBEV strains in relatively small geographic areas (*25*–*28*). For example, TBEV isolates from southwestern Germany were closely related to strains from the Czech Republic, Austria, Switzerland, Slovakia, and Italy (*28*). In addition, the clustering of whole-genome sequences from Dronten with strains from Germany and of those from Hellendoorn with a whole-genome sequence recently reported from England could be in line with the recent westward spread of TBEV in Europe (*29*,*30*).

The diversity of TBEV variants in both the Netherlands and England points toward multiple introduction events in both countries, possibly through migratory birds (*31*). Migratory birds have been implicated in the spread of TBEV before (*25*,*29*,*32*). However, additional whole-genome sequences are needed from other TBEV risk areas from Europe for a more complete phylogenetic and phylogeographic analysis to determine the mechanisms of spread of the virus. Also, it remains unclear which TBEV strain circulates in the province of Noord Brabant, where 3 human TBE cases have recently occurred, or the 2 nature areas, Rijk van Nijmegen and Natuurpark de Leemputten, where we detected TBEV RNA in rodents but could not perform detailed phylogenetic analyses because sequences were too short.

As found elsewhere in Europe, TBEV in the Netherlands appears to have a rather focal distribution. For example, we found TBEV RNA–positive ticks in the Utrechtse Heuvelrug in 2018 and 2019 at the exact same location but not elsewhere in this relatively large nature area. Moreover, it appears that the virus might locally disappear. For example, we did not find any TBEV RNA–positive ticks in the Nijverdal garden, despite a remarkably high infection prevalence in 2017 (1/63 ticks) and 2018 (1/92 ticks) (*9*). Although absence of evidence is not evidence of absence, we thoroughly sampled the entire garden and collected 1,194 questing ticks during 3 sampling events in April, May, and June 2019. Although 1 of the rodents collected from this location in May 2019 was seropositive, this result could not be confirmed by an SNT because of insufficient serum volume. Experimental studies have shown that wild rodents mount a strong antibody response to TBEV that can still be detected at 168 dpi (*33*). Therefore, this animal could have been exposed to infected ticks in late 2018 rather than 2019. Local fade-out of TBEV in former transmission areas has also been reported elsewhere in Europe, including Germany (*34*), Denmark (*35*), and France (*36*), so this phenomenon appears common across a wide diversity of habitats. However, TBEV also reemerged in some of these areas, raising questions as to whether the virus was reintroduced (e.g., by migratory birds) or had actually persisted below levels at which it could be detected (*37*).

Our very large sampling effort of >46,000 questing ticks but low number of TBEV RNA–positive pools (n = 7, representing 137 individual specimens) underlines the challenges of using tick surveillance to identify TBEV risk areas (*38*,*39*). Instead, screening of humans and sentinel or reservoir hosts might provide a more effective indicator (*38*,*40*–*42*), although these methods also have their drawbacks (*39*). For example, a recent serologic survey of employees and volunteers of nature management organizations in the Netherlands found a seroprevalence of 0.5% (3/556; 95% CI 0.1%–1.6%) among participants (*43*). Although all seropositive participants had worked in provinces with confirmed cases, precise source attribution is difficult. Likewise, serologic surveillance of large sentinel hosts such as roe deer can only indicate past exposure to TBEV, and their relatively wide foraging range (≈51–136 ha) (*44*) hampers precise identification of TBEV foci (*39*). Moreover, cross-reactivity between different flaviviruses is well documented and might lead to false-positive results in both humans (e.g., in case of yellow fever vaccination) and sentinel hosts (e.g., when other flaviviruses circulate in the environment) (*45*), requiring SNT for confirmation. In contrast, wild rodents are natural reservoir hosts that develop levels of viremia high enough to demonstrate active TBEV circulation (*46*,*47*). Moreover, rodents have small home ranges (<0.5 ha in forest habitats) (*48*,*49*), which enables more accurate identification of foci (*39*). On the other hand, catching infected rodents during the small window of viremia is challenging, and cross-reactivity of flaviviruses remains an issue. Moreover, sampling a sufficiently large number of wild rodents to detect TBEV foci is a considerable endeavor that also poses ethical questions, such as potential impacts on local populations of *A. flavicollis* mice, still a relatively rare species in the Netherlands. Given that the spatial distribution of TBEV appears to be increasing in the Netherlands but that the minimum infection prevalence in ticks is extremely low (0.02% vs. 0.1%–2.7% elsewhere in Europe) (*39*), we suggest continued monitoring using an integrated approach that combines passive surveillance of humans and sentinel hosts such as game animals (e.g., deer) to detect potential TBEV risk areas, after which more targeted local screening of rodents and ticks may confirm actual virus circulation.

The mechanisms underlying the sustained circulation of TBEV in the Netherlands are unclear. Nonsystemic virus transmission from infected nymphs to uninfected larvae during simultaneous feeding on rodent hosts (cofeeding) is considered a prerequisite for endemic circulation of TBEV (*7*,*10*). Northwestern Europe was thought to lack the specific climatic conditions required for cofeeding transmission, and past modeling studies had therefore predicted that TBEV would not become established in this region (*10*). Nonetheless, we found cofeeding of larvae (n = 228) and nymphs (n = 27) on 21 (6.6%) of 320 rodents and in 8 of 13 locations. These findings suggest that cofeeding is a potential route of transmission in the Netherlands. Although none of the feeding ticks were TBEV RNA–positive, this finding might be explained by low sample size. Previous work showed that cofeeding also occurred on 3.6% of rodents in England (*50*). Past models might have accurately predicted TBEV foci in Central Europe based on climatic data (*10*), but the presumed underlying relationship cannot explain TBEV circulation in Northwestern Europe. Given the recent emergence of endemic foci in Northwestern Europe and the occurrence of cofeeding in this region, the distribution of TBEV will likely continue to change. Future studies should investigate how common cofeeding is in areas where TBEV does not circulate and identify the ecologic conditions that promote the synchronous activity of larvae and nymphs in emerging areas.

In summary, we found TBEV RNA in rodents and tick pools from 5 foci in the Netherlands and that 3 different variants of the TBEV-Eu subtype are currently circulating, suggesting multiple introductions. Our findings, along with other human cases outside known TBEV hotspots, show that the distribution of TBEV is more widespread than previously demonstrated in this country.

Appendix 1 Additional information about analyses conducted in study of continued circulation of tick-borne encephalitis virus variants and detection of novel transmission foci, the Netherlands

Appendix 2 Additional data used in study of continued circulation of tick-borne encephalitis virus variants and detection of novel transmission foci, the Netherlands
