# Supplementary material for: Continued Circulation of Tick-Borne Encephalitis Virus Variants and Detection of Novel Transmission Foci, the Netherlands
Source: Emerg Infect Dis. 2022 Dec;28(12):2416–24. doi: 10.3201/eid2812.220552 (PMC9707572; doi:10.3201/eid2812.220552)
Supplement: Appendix 1 — Additional information about analyses conducted in study of continued circulation of tick-borne encephalitis virus variants and detection of novel transmission foci, the Netherlands [file 22-0552-Techapp-s1.pdf]

Article DOI: https://doi.org/10.3201/eid2812.220552

Continued Circulation of Tick-Borne Encephalitis Virus Variants and Detection of Novel Transmission Foci, the Netherlands

Appendix 1

Species Identification of Ticks Collected from Rodents

Larvae were placed in Lysis Matrix D tubes and nymphs in Lysis Matrix Z tubes (MPBio) containing 500 µl of Dulbecco Modified Eagle's Medium (DMEM). Samples were homogenized using a FastPrep-24 Sample Preparation System (MPBio). Tubes were subsequently centrifuged at maximum speed for 5 minutes and 100 µl of supernatant was added to 100 µl of MagNA Pure External Lysis Buffer (Roche). Each sample was spiked with a known amount of internal isolation control (Phocid Distemper Virus; PDV, Ct 30). Total nucleic acid (TNA) was extracted using the EZ1 Nucleic Acid Automated Purification System (Qiagen) according to the manufacturer's instructions. TNA was tested in TaqMan RT-qPCR using the qScript® XLT One-Step RT-qPCR ToughMix Low ROX (Quantabio) and using primer and probe sets (at 10 µM and 5 µM, respectively) targeting the internal transcribed spacer 2 gene (*ITS2*) of *I. ricinus* and *I. trianguliceps* (Appendix 1 Table), the 3′ untranslated region (UTR) of TBEV (*1*), or the PDV hemagglutinin (H) gene (*2*) (Appendix 1 Table). The primers specific to *I. ricinus* and *I. trianguliceps* were designed within the *ITS2* region of the nuclear rDNA transcriptional unit using sequences obtained from GenBank.

Serologic Detection in Rodents

Blood samples taken from rodents were collected in MiniCollect Blood Collection tubes (Z serum separator) (Greiner-Bio), allowed to clot at room temperature for several hours, after which serum was separated by centrifugation at 2100 × g for 10 min. Serum was stored at −20°C until further analysis. For serologic analysis, serum samples were diluted 1:50 in phosphate buffered saline (PBS) and incubated on precoated ELISA plates for 1 hour at 37°C. Wells were

washed five times with wash buffer. Next, 100 µl of Conjugate was added to each well and incubated for 1 hour at 37°C. Wells were washed five times and subsequently developed using 100 µl of TMB-Complete. After the addition of Stop Solution, plates were read at an optical density ratio (OD) of 450nm and samples with a value of at least three times the OD of the negative control (3×OD_{NC}) were considered as seropositive. Samples with a value between two and three times the OD of the negative control (2×OD_{NC}) were considered borderline.

Appendix 1 Table. Nucleotide sequences of primers and probes used for the detection of *Ixodes ricinus*, *Ixodes trianguliceps*, tick-borne encephalitis virus (TBEV), and phocid distemper virus (PDV, internal control)

Target	Name	Sequence
I. ricinus, internal transcribed spacer 2 (ITS2)	#539_Ix_ri_ITS2_Fw	CGA-AAC-TCG-ATG-GAG-ACC-TG
	#540_Ix_ri_ITS2_Rv	ATC-TCC-AAC-GCA-CCG-ACG-T
	#566_Ix_ri_ITS2_Pr (Cy®3-BHQ1)	TTG-TGG-AAA-TCC-CGT-CGC-ACG-TTG-AAC
I. trianguliceps, ITS2	#548_Ix_tr_ITS2_Fw	GTCGTTGGGTTTGCTTCCTA
	#549_Ix_tr_ITS2_Fw	GTAGACGTCCTCGCTTCCAC
	#550_Ix_tr_ITS2_Pr (JOE-BHQ1)	CCGATGAATACTGGAGCCAT
TBEV, untranslated region (UTR)	#111_TBEV_UTR-F-3	GGG-CGG-TTC-TTG-TTC-TCC
	#112_TBEV_UTR-R-3	ACA-CAT-CAC-CTC-CTT-GTC-AGA-CT
	#113_TBEV_UTR-Pr-3 (FAM-MGB)	TGA-GCC-ACC-ATC-ACC-CAG-ACA-CA
PDV, hemagglutinin gene (H gene), internal control	#483_PDV_Fw	CGG-GTG-CCT-TTT-ACA-AGA-AC
	#484_PDV_Rv	TTC-TTT-CCT-CAA-CCT-CGT-CC
	#485_PDV_Pr (TAMRA-BHQ2)	ATG-CAA-GGG-CCA-ATT
TBEV, Envelope protein	TBEV-E_Fw	GTG-GGA-AAC-AGG-AAG-GCT-C
	TBEV-E_Rv	CCA-CCC-TGG-TGT-TCT-TCA-G

References

1. Schwaiger M, Cassinotti P. Development of a quantitative real-time RT-PCR assay with internal control for the laboratory detection of tick borne encephalitis virus (TBEV) RNA. J Clin Virol. 2003;27:136–45. PubMed https://doi.org/10.1016/S1386-6532(02)00168-3

2. van Doornum GJ, Schutten M, Voermans J, Guldemeester GJ, Niesters HG. Development and implementation of real-time nucleic acid amplification for the detection of enterovirus infections in comparison to rapid culture of various clinical specimens. J Med Virol. 2007;79:1868–76. PubMed https://doi.org/10.1002/jmv.21031
